# The co-constitution of the self and the world: action and proprioceptive coupling

**DOI:** 10.3389/fpsyg.2014.00594

**Published:** 2014-06-12

**Authors:** Olivier Gapenne

**Affiliations:** CNRS, BioMécanique et BioIngénierie, UMR 7338, Université de Technologie de CompiègneCompiègne, France

**Keywords:** proprioception, sensory substitution, enaction, perception, coupling, self-world duality, cybernetics

## Abstract

This article proposes a theoretical reflection on the conditions for the constitution of a distinction between the self and the world by a cognitive system. The main hypothesis is the following: proprioception, as a sensory system that is habitually dedicated essentially to experience of the body, is conceived here as a coupling which is necessary for the dual and concomitant constitution of a bodily self and of a distal perceptual field. After recalling the singular characteristics of proprioceptive coupling, three lines of thought are developed. The first, which is notably inspired by research on sensory substitution, aims at emphasizing the indispensable role of action in the context of such perceptual learning. In a second part, this hypothesis is tested against opposing arguments. In particular, we shall discuss, in the context of what Braitenberg called a synthetic psychology, the emergence of oriented behaviors in simple robots that can be regulated by sensory regulations which are strictly external, since these robots do not have any form of “proprioception.” In the same vein, this part also provides the opportunity to discuss the argument concerning a bijective relation between action and proprioception; it has been argued by others that because of this strict bijection it is not possible for proprioception to be the basis for the constitution of an exteriority. The third part, which is more prospective, suggests that it is important to take the measure of the phylogenetic history of this exteriority, starting from unicellular organisms. Taking into account the literature which attests the existence of proprioception even amongst the most elementary living organisms, this leads us to propose that the coupling of proprioception to action is very primitive, and that the role we propose for it in the co-constitution of an exteriority and self is probably already at work in the simplest living organisms.

## INTRODUCTION

Inspired by the conjunction between the traditions of constructivism and phenomenology, which has been formulated and elaborated recently in the framework of the paradigm of enaction (Varela et al., 1991), this article proposes a reflection on the conditions for the constitution of a double perceptual polarity: that of the self (mainly a bodily self here), and that of a structured exteriority. In other words, how it is that a cognitive agent manages to constitute a “referential impression” of the lived world at the same time that it specifies itself. This constitution, or the genesis of a structured experience, comprises two aspects: the first concerns the fundamental properties of the objects that are co-constructed (self and/or world), such as substantiality, distality, figurability, tangibility, or yet again a sense of sameness; the second concerns the properties of the perceptual field itself as well as its englobing character (the fact that the agent experiences the feeling of being inside). We will not here exhaustively address all these properties. Rather, we propose to focus on the *initial* and *generic* conditions for this constitution of an organized process of appearing: firstly at the level of perceptual consciousness; and then at the level of a generalizing, imaginative, and anticipatory consciousness. First of all, we will recall the importance of “bodily action” as action produced by an agent, and inducing sensory effects at the level of the same agent. This activity, conceived as sensory-motor or kinesthetic coupling, characterizes the concrete and continuous mode of relation that the agent entertains with its body and its environment (the dimension of what is present). The role of this coupling is to introduce a necessary variation which will form the basis for an activity of synthesis which will allow not only for feeling but also for the appearance of objects. A reminder of the situation of sensory substitution will serve as an example for this aspect.

Then – and this will be at the heart of this article – when one wishes to account for the constitution of the distinction between the self and the world, there is a necessity for the acting agent to make a distinction between two sources of variation in the sensory signals that affect it: those that are related to its own activity, and those that arise from the environment (considering that the perceived organization of this environment is not pre-defined). We may note that an absence of distinction, or a confusion, between these two sorts of signal directly threatens the agent since it favors the constitution of erroneous perceptions which may be deleterious, and are at the very least unsettling as in the case of illusions of vection and self-motion. Thus, going further toward a definition of the mechanisms of the constitution of this phenomenological dissociation between self and world, we propose a mechanism of “filtering and calibration” which allows an agent, when its sensory organs are submitted to variations in their states, to be able to attribute these variations either to its own activity (and thus as effects of its actions), or to events over which it has no control. In this way we develop the hypothesis, following on from the reflections of Poincaré (1902, p. 84) on the construction of perceptual and conceptual space, that the singularity of proprioception lies in the fact that it is a firm reference-point which enables this process able to play this role of “filtering and calibrating” (Declerck and Gapenne, 2009; Gapenne, 2010a,b; Blanchard et al., 2013). This will lead us to reflect upon the organized behaviors of certain artificial agents which do not possess proprioception, and to critically discuss theses which claim that it is possible to constitute spatiality solely on the basis of external sensory inputs.

Finally, in conclusion, we will redefine action as not being limited to the motor dimension of effective action, but as deriving from an organization where performance and sensation are necessarily coupled, and to postulate that motor-proprioceptive coupling plays a foundational role in the construction and the genesis of the enactive process of partitioning the self and the world, the inside and the outside, and so on.

## ACTION-SENSATION COUPLING IN SENSORY SUBSTITUTION

Amongst the various fields of research which have confirmed the importance of embodiment and action, as starting-points for the constitution of a process of appearing, the work of Bach y Rita in the 1960s on sensory substitution by means of a special device (TVSS: tactile vision sensory substitution) holds a prime place (Bach Y Rita, 1972). By actively using this device (see below for a detailed description), blind or blindfolded participants are able to perceive distal events (the position and the form of a 3D object) as in vision and to improve significantly their performances (discriminate objects in a scene and manage the interposition) by learning. Right from the start, many authors such as Paillard (1971) did not fail to emphasize the interest of this work, which opens up the possibility for the precise experimental study of the genesis of a form of perception which derives from action. This study appeared all the more original in that it mobilized proximal sense-organs (in this case, tactile sensor) in the constitution of an experience of an object at a distance without any direct contact. Although many summaries of these studies have already been published (e.g., Kaczmarek et al., 1991), we consider that it is useful to reformulate here the principle of sensory substitution. Technically, sensory substitution requires the insertion of an activator or stimulator (or a whole set of activators or stimulators) as an intermediary between two sensory systems, one artificial and the other natural. In other words, the “substitution” involves a doubling of the stimulation and thus a doubling of the transduction^1^: an artificial transduction (via a sensory device = transduction 1), and a natural transduction (via a functional sensory system = transduction 2). This double transduction, via the insertion of an artificial captor and activator, makes it possible to provide access to a sensory flow which would not be available without this technical mediation. In the pioneering work of Bach-y-Rita et al. (1969) on the TVSS, it was a question of providing blind persons with access to an optical flow, via a camera (transduction 1), with electro-mechanical stimulators which relayed the signal from the camera and stimulated natural sensory organs which were available, i.e., the tactile receptors of the skin (transduction 2). As can be appreciated immediately, and as many subsequent developments have shown in practice (for relatively recent reviews see Wall and Brewster, 2006; Visell, 2009), this principle of substitution can theoretically substitute any sort of flow by any other (visual–auditory, auditory–tactile, tactile–tactile, etc).

However, the use of these instruments rapidly revealed that the substitution is not limited to this double transduction in the sense of a two-stage transfer of input signals to the nervous system. Firstly, it is imperative that the signals that are transmitted should be subject to variation. Secondly, and this is the really essential point, the “substitution” only becomes effective if this variation is amenable to interpretation; and the key condition for this is that the variation in question should be determined by the user. It must therefore be well understood that the constitution of the properties, and in particular the spatial properties of the flow that is substituted (for example, vision being substituted by the tactile modality) does not derive from simply capturing the spatiality inherent in the organization of the network of activators which deliver the signals (for example, a square 20 x 20 matrix of 400 activators in the case of the TVSS). In this sense, the logic of the constitution of perceptual experience, and more generally of cognitive experience, via this type of device cannot be limited solely to the double transduction of signals whose variation arises from external events. This variation must be an *active* variation, i.e., the variation must be produced and controlled by the agent. Thus, the “substitution,” as a process which is equipped, must also include the tool of an* inverse* double transduction corresponding to the action produced by the body with respect to the instrument, an action producing a movement of the instrument with respect to the environment.

It is thus essential to understand that the “substitution” cannot be solely sensory; this has led us to propose that the substitution is rather perceptual, in the sense that it involves a moto-sensory^2^ coupling whose closure is ensured by the technical system on one hand and the user on the other. In addition – and this is in a way a consequence of the preceding point – the process involved is not properly speaking a “substitution,” but rather what we have called a “supplementation” (Lenay et al., 2003), this latter term being conceptually more adequate. And as a matter of fact, the system that is called “tactile–visual substitution” does not give rise to a truly *visual* experience as such. The instrument, in particular when it is actively taken in hand, opens up an unprecedented space of experience, which makes it possible to interpret certain properties of the “novel” moto-sensory flow. And in the case of the TVSS, it is remarkable that the instrumented activity makes it possible to interpret distal spatial qualities on the basis of proximal tactile signals. Guarniero (1974) evidences that after several hours of use, a blind user is able to recognize simple objects at a distance, including moving objects, and to interpret certain events as interpositions.

A final point that is worth mentioning is that the stimuli delivered by the tactile stimulators are not forces of a sort which would constrain the movements of the subject; this is in contrast to devices such as the robotic arm PHANToM Desktop. With the TVSS, the stimulation consists of a pressure on the skin, but it does not deliver a return of effort of a kind which could guide the movement. This is an essential point because, although it involves a tactile activator, the TVSS is an interface which is “gestural,” and in this sense much closer to visual gestures. Indeed, the movements of the ocular globe are produced without any constraint from the optical flow, since this flow does not deliver any forces such that the movement of the ocular globe would be mechanically affected and guided. In other words, the tactile stimulations of the TVSS do not directly constrain the movements of the agent. Thus, in the two cases, the control of the movement must be actively produced by the agent – and this is a quite general situation. In this context, a gesture (an organized exploratory movement) can be minimally described as an attractor where each state must be defined by at least two parameters: a definite position of the point of action in (x, y, z) co-ordinates; and a value of the sensation (0 or 1) indicating the absence or presence of an event in the environment. The temporal succession of these states (x,y,z,e) describes a trajectory that we may define as a “gesture,” or alternatively as a “strategy” (Stewart and Gapenne, 2004). In this situation, what the subject receives at each point in time is just a sensation (or a set of sensations), and the mere projection of this sensation onto the sensory organ is not sufficient to initiate perceptual activity. If the subjects do succeed in perceiving “objects,” it can only be through their active exploration, and by integrating over time their movements, the tactile sensations, and their kinesthetic sensations. Thus, the situation of perceptual supplementation is exemplary because, quite besides the technical innovation, it makes it possible to re-create at a micro-developmental scale a situation of perceptual learning. Even though this learning does not have exactly the same meaning for an adult and for a newborn child, we can nevertheless follow through the necessary steps for the mastery of a new mode of coupling.

In another technical context, inspired by the work of Meijer (1992), Auvray et al. (2005, 2007) has proposed a description of the steps involved in the appropriation of a device by sighted adult subjects. Without going into the fine details of the succession of all these stages, let us consider the first two which are of particular interest here. The first stage is called “contact”; it involves learning the sensory-motor regularities necessary to stabilize and to actively maintain perceptual contact with the stimulus. As for the second stage, labeled “distal attribution,” it corresponds to understanding the origin of the sensations as deriving from the fact of making contact with an object situated in the perceptual space opened up by the tool. This second stage is perhaps unfortunately labeled, since it risks confusing the fact that the variation in the sensations has an origin which is *distinct* (i.e., not related to the determination of my actions) as compared to an origin which is *spatially distant*, which is of course not the same thing. In this situation, as in the original experiment of Epstein et al. (1986), the participants using a sensory substitution device but not being informed about its functioning are asked for the nature of what they perceived and had to make a choice among several scenarios (e.g., “*sensors, located on my head and hand, record the locations of my head and hand and produce different stimulation intensity levels whenever those locations change.”* or “*a camera, located in front of me, detects both hand and head movements and sends a signal to the device whenever movement is initiated.*”) that proposed a rationale for what was happening. The point of interest is that the subjects produce sensory variations as a result of their own movements; but, taking into account the fact that the subjects are ignorant as to the experimental setup, the situation remains somewhat ambiguous so that the interpretation of the variation in the stimuli is not necessarily that of a determination through agency. And even when it is, the subjects have great difficulty in considering that the source of these variations may be external and distant. It is clearly apparent that whereas at the stage of contact the subjects often succeed, in the experiments of Epstein et al. (1986) and Auvray et al. (2005), in expressing their consciousness of the relation between their actions and the reafferent sensations, this is because the source is fixed and cannot produce a stimulation unbeknown to the subject if the latter is immobile and not stimulated. Nevertheless, the sensitivity to the spatio-temporal coincidence between the movement and the tactile reafference does not seem to be so obvious to all the subjects. This point is important, since it indicates that even in such favorable conditions the interpretation in terms of agency is not guaranteed with an external source, and it is necessary to introduce certain conditions of manipulating the coupling (for example by giving the possibility of interposing a screen between the sensory captor and the source) in order to lift the ambiguity (Auvray et al., 2005).

To sum up this section, and referring to the work on sensory substitution, we will note three main points. Firstly, *modulo* the necessary movement by a suitably equipped agent, it is possible to constitute a distinct, distal appearance. Secondly, this appearance is not reducible to an analysis of the tactile sensations or of the movements produced in order to determine them; in both cases, the tactile and kinesthetic sensations are “forgotten” and replaced by a consciousness focused on the events in the environment. Thirdly, if the subjects are not informed about the properties of the coupling system (for example the TVSS), and are not informed about what there is to be perceived by specifying explicitly that the source is clearly positioned “out there” at a distance, it seems that the experience of agency is not guaranteed. This being so, with respect to our question concerning the constitution of the self/world distinction, the analyses which have been carried out so far by means of the experiments of sensory substitution/perceptual supplementation only provide us with partial answers as to the conditions of this constitution.

One of the reasons for this is that the studies in this domain have been concerned above all with the constitution of the “object” pole; the other pole, that of the “subject,” is referred to an implicit, pre-reflexive register which at best plays a role of motivation, and not really of exposition (Husserl, 1989). It is to be noted immediately here that the problem arises from the impossibility of having simultaneous experience of the two poles; in fact, certain studies have clearly shown that it is possible to have recourse to the principle of sensory substitution in order to constitute/recover bodily experiences (Tyler et al., 2003). In line with this, the “subject” pole can also be recovered in this way, to the extent that it refers to a kinesthetic, bodily experience. In this light, we come to realize that there is a point which has remained obscure in all these analyses: and this is, to understand *how* the sensory flow generated by the active movement by the agent, which determines the variation in the flow, can actually be partitioned by the agent. One way to lift the veil of mystery would be to consider that the deployment of each movement is always associated with a *double* reafferent flow (here, a tactile flow and a proprioceptive flow). One of these reafferent flows (the tactile flow) would be contingent, and the other one (the proprioceptive flow) would be absolute – at least to a first approximation. The hypothesis would then be that the proprioceptive system contributes to a filtering, since it provides the agent with a non-ambiguous indication as to whether he/she is active or not. We shall develop this hypothesis and make it more precise in the next section. In order to close the present section, we will remark again that if the tool can be “forgotten” when it contributes to the accession to an experience of the self and/or an exteriority, this “forgetting” also concerns the tactile sensations as such. It would seem interesting to delve more deeply into this “disappearance from experience” which occurs at the level of receptors such as the retina or the cochlea. In the case of a prolonged and intensive use of the TVSS, would one arrive at a stage when the tactile sensations would have become just as inaccessible as retinal sensations?

## THE SINGULARITY OF PROPRIOCEPTION

In the matter of proprioception, it is important to be very precise (Stillman, 2002). Only too often, and wrongly, “proprioception” is misleadingly over-represented as the perception of self as an embodied, acting agent. But it is obvious, as indicated by the unfortunate expression “proprioceptive function” as coined by Gibson, that this sort of perception of bodily activity and the self involves many (and indeed, *a priori*, all) perceptual systems. For this reason, rather than the term “proprioceptive function,” we prefer the term “kinesthetic function” which does properly refer to the multimodal experience of the body at rest or in movement, static or dynamic. We will reserve the term “proprioception” as one specific perceptual system among others, which is indeed involved in the experience (and the regulation) of movement, posture and balance; but as we shall see, proprioception also does more than this. Anatomically, the proprioceptive system mobilizes sensory organs, afferent innervations, and specific cortical structures which are known in part today (McCloskey, 1978; Hogervorst and Brand, 1998; Romaiguère et al., 2003). A notable feature of this system is that all the sensory organs are localized in the core of effectors (muscles, tendons, articulations) involved in the maintenance and the animation of the skeleton. It is thus a case not just of relative proximity, but of genuine contiguity between the sensory organ and the effector. It is thus important to understand that variation in the activity of the proprioceptive organs (neuromuscular spindles, neurotendinous organs, or articulatory receptors), variation which is necessary for them to function, is intimately related to variation in the activity of the effector itself. This has led proprioception to be called “muscular sense” ever since its first description by Bell (1826), including mainly a sensitivity to movement and to position. The specificity of proprioception derives from the fact that all the other sensory organs respond to variations essentially linked to mechanical, chemical, optical, or other flows which come from the environment. More precisely, since a living organism is never completely static (physiological tremor, ocular micro-nystagmus), sensory organs can receive variations in input whose amplitude cannot be directly related to the amplitude of movements of the agent (Lockhead, 1992). The crucial point here is thus that variation in the stimulation of all the sensory organs, with the sole exception of proprioception, is linked to bodily engagement but is always liable to be compounded with the effects of events external to the agent. In other words, variation in the activity of the sensory organs, which is itself linked to variation of their source, is always potentially composite and ambiguous quite simply because the source of this variation is potentially dual (and, in the event, almost always is dual since there is a mix of variation due to the agent itself and variation due to external events). The proprioceptive system thus has this prime singularity, that it is always activated by deformations of the body, and (in natural conditions) by nothing else. As experimental studies in humans and animals have already suggested empirically, the consequence of this is that if proprioception plays an unquestionable role in the perception of bodily events (Farrer et al., 2003), it can also play a role in the perception of external events and, more fundamentally, in the genesis of the perception of such events (Buisseret et al., 1988; Roll et al., 1991). We therefore suggest that by having at its disposal a moto-sensory system strictly associated with its own activity, the agent possesses a powerful tool for filtering and calibrating signals for which it does not control the determinism.

Philipona et al. (2003), far from any immediately phenomenological considerations, have taken up this line of argument on a strictly formal basis, and have proposed an algorithm, based on inputs and outputs, which is apparently able to deduce the geometry and the dimensions of an external space without any *a priori* knowledge. The calculating artifact (a virtual poly-articulated robot) has at its disposal input signals coming from two types of sensors: sensors which are sensitive to changes in the positions of the articulated segments of the robot, and sensors which are sensitive to the presence of light in the unknown virtual environment. In addition, effector organs controlled by the algorithm produce the movements of the robot. In the first instance, the algorithm distinguishes two types of signal according to whether they are related to its own movements or not. In other words, the algorithm can discriminate between exteroceptive signals which are produced when the robot is static, and proprioceptive signals which possess a bijective relation with its own movement (“certain inputs react always in the same way to motor command,” Philipona et al., 2003, p. 3). Then, in a second instance, the robot (or rather its “brain”) is able to distinguish between two sorts of exteroceptive signals: *exafferent* signals which are independent of its own movement (when the robot is static and the sensors are subject to variations), which enables the induction of a vector called “*representation of the state of the environment”*; and signals which are associated with movements of the robot (whose sensors are subject to variations related to *reafferent* signals), which enables the induction of a vector called “*representation of the exteroceptive body.”*

Now although this study does have the interest of proposing a possible mathematical formulation of the distinctions which an organism can make in order to perceive itself as different from its environment, it has serious limitations. In particular, it does not treat the case of a double source of stimulation when the robot is in movement (a *combination* of exafferent and reafferent signals), which is in the end the crucial situation for a living organism, and which is at the core of the dilemma we have to deal with. Moreover, the relation between proprioception and exteroceptive reafferents is envisaged merely as a possible intersection. From our point of view, the constitution of a genuine process of appearing (i.e., the microgenesis of perception) requires a genuine articulation, and not just a contingent intersection between entities that are presupposed to be distinct. Finally, besides the hypothesis of a bijective relation between action and sensation in the case of proprioception (see below), and its limitation in this model to a capture of position (even if movement and position are to some extent correlated), the hypothesis that the motor commands – which will prime the moto-sensory coupling and thus prime the subsequent inferences realized by the “brain” – are produced “at random” remains mysterious. Where do these commands come from? Why do they take the form that they do? Are they generated by a “program”? As I will say below, this conception of commands as pure effectuation does not seem adequate in the case of living organisms.

A second singularity of proprioception is that these sensors do not seem to be submitted to the activity of an efferent sensory system, as are all other sensory systems (e.g., Warr, 1975). In addition, the activity of proprioceptive receptors does not seem to be modulated by anything other than the activity of the effectors to which they are linked. The receptors or the primary and secondary sensory nerve-endings situated in the equatorial zone of the muscular fibers present a variation in their potential as a function of the modulation of the tension of the muscular tension. And even in the case of the gamma loop, the neurons emanating from the anterior horn of the spinal cord are moto-neurons which modulate the stretching of the fibers, but they are in no way sensory efferent fibers which modulate the activity of the sensory nerve-endings themselves. This anatomical particularity has functional consequences. The activation of afferent proprioceptive fibers can modulate the behavior of the receptors of other sensory systems via their action at the level of central nuclei from which efferent fibers leave toward the other sensory receptors. Conversely, the other sensory systems are not able to carry out such a modulation, other than indirectly via the modulation of the tension of the muscular fibers.

## THE ABSENCE OF PROPRIOCEPTION AND THE BIJECTION ACTION/PROPRIOCEPTION

In order to discuss the theoretical proposition formulated above concerning the role of proprioception, and maybe to contest it, we shall now consider two arguments which go against it: one of these argument is empirical and factual, and the other is theoretical. The first argument refers to the possibility of producing spatially organized behavior without any recourse to proprioception; the second posits that the constitution of a space is impossible if it is admitted that the relation action-proprioception is bijective.

In his essay in synthetic psychology, Braitenberg (1986) presents some very simple robotic architectures based on direct connections between sensors and effectors, which are nevertheless sufficient for the mobile robots to exhibit distinctive behaviors, such as attraction and repulsion, with respect to a source. At no point in his short and fascinating text does Braitenberg even so much as mention the very idea of proprioception – which leads him, in fact, to put forward some very internalist and representationalist propositions. We may recall here that the famous “tortoises” of Gray Walter (Machina Speculatrix) were likewise bereft of any proprioception (they possessed only a shock-sensor), and were already able to exhibit behavior such as “return to the nest,” an “attractive” site where the tortoise could recharge its energy; this site possessed a light which served as an external source for guiding the tortoise. Let us consider then this case of a displacement toward a source of light. The robots were equipped with a photo-electric cell (a photo-sensitive sensor); detection of the light was supposed to produce exploratory movements which here were of two types, “translation” or “rotation.” The composition of these two sorts of movement produces a sinusoidal (or ellipsoidal) trajectory, whose amplitude theoretically tends to decrease as the robot approaches the source. What can we learn from the emergent behaviors produced by these automata? It is clearly a case of emergence, in the sense that the trajectory produced by the agent, and described by the observer, is in no way programmed as such (even though it results from the operation of an electronic circuit), and it is not learned. These behaviors demonstrate that an agent, even an artificial agent, can produce spatially organized behavior without any recourse to “proprioceptive” signals concerning its own material architecture and its own movement. This self-organization does however, have some limits, in particular concerning the choice of the material architecture and the possibilities of action which are associated with it. We may note that, unlike the virtual robot of Philipona et al. (2003) described above, these robots do not have any proprioception and so the problem of “partitioning” simply does not arise. Moreover, the problem of portioning “external” signals as arising from the movement of the agent versus that of the environment cannot be resolved by intersecting external and proprioceptive flows of sensation. So what, after all, does this tropism toward a light-source tell us? It indicates that the action of the agent (the activation of a motor producing the rotation of the wheels) can be controlled by the capture of a contingent “external” signal on which feedback is applied. But then, with respect to our hypothesis concerning the deleterious consequences of confusion concerning the source of variation, why in the case of these robots does this not cause totally aberrant behavior? When the photo-electric cell is activated, the robot cannot “interpret” this activation as being necessarily related to its own rotation (the light-source is fixed), because it does not have any signals concerning its own movement. So what could possibly constitute a “pathological” behavior in this case? This strictly external guidance of the actions which are successively produced rests on the tolerance of a fusion of the sources of contingency: the light-source can be displaced by the experimenter, or the movement of the robot can produce a displacement of the sensor, such that it is no longer in phase with the source. And in fact, an examination of the concrete situations reveals that the regulation occurs in the succession of these two modes of variation, and does not tolerate well their concurrence. However, and this is a key point, the great majority of natural situations do expose the agents to the simultaneity of the variations.

Of course, this tropism toward a light-source is reminiscent of the way bacteria climb a glucose gradient; we will come back to this point, to suggest that the management of this simultaneity by a living organism is not of the same order as the Braitenberg robots, and as in the case of micro-organisms, does not need a central nervous system to be achieved.

The argument concerning the bijection action-sensation is in a way the counterpoint to the preceding question. If one admits the existence of an agent which would possess only proprioception, such an agent would not be able to have access to any variations other than those produced by its own actions, and it would therefore be in a situation where the variations are totally determined (Piaget, 1937; Lenay, 2006). In this case, no opening toward the exterior would be possible, and neither would an access to the bodily self on the basis of the actual variations. This argument is often invoked, on the one hand to affirm that proprioception alone, in and of itself, cannot open the way to spatiality; and on the other hand, it constitutes a risk of a return to a representationalist conception of bodily experience. Both of these risks are real. However, this hypothetical situation and the associated risks should be put in due perspective. Firstly, there is no known living organism whose organization is founded strictly and solely on proprioception. All known living organisms do have two sorts of sensors, those that are proprioceptive, the others which are sensitive to events which are totally or partly independent of the actions of the organism. The question is thus not so much that of a total determinism of the moto-proprioceptive loop, but rather that of the articulation between this loop and the others. Secondly, one can question the status of a possible bijection; and also ask questions about the bijection itself. If the hypothetical bijection supposes that the motor command, specifying a precise value for a parameter of position, speed or other, has the effect of producing a corresponding unique value at the level of the sensor, this supposition postulates anew that the command/action is a matter of pure effectuation, and tends to deny the importance of the differential of the activity of the sensor. As for the bijection itself, it may be doubted whether it could ever actually be realized, not only because the bandwidth for proprioceptive sensors is limited and their response not so reliable (Wann and Ibrahim, 1991), but also and above all because of the principle of functional ambiguity which refers to the radical impossibility for a command to totally anticipate the concrete realization of the action. In particular, gravitation and friction always leave a certain degree of uncertainty concerning the movement which will actually occur. These variations, which cannot be determined by the command, are actually a condition for the possibility of constituting an experience of the body/self – even if, as we have already said, this kinesthetic experience involves the set of sensory organs as a whole.

## LIFE AND THE SELF-WORLD DUALITY

In this article we have proposed that the constitution of an experience of the distinction between the self and the external world supposes that the agent has at its disposal a way of coupling its means of action and its means of sensation; the latter being sensitive to variations in the signal that are related, or not, to the effects of actions produced by the agent itself. We have also postulated that moto-proprioceptive coupling plays a decisive role in this constitution, to the extent that it allows for the advent of a referent with respect to which other sensory signals can be sorted and calibrated. We have insisted on this function of sorting, because it seems to us to be indispensable, *via* action, in the constitution of two distinct poles of experience, that of the subject and that of the object. On this point, we wish to draw attention to the fact that even the simplest forms of life (even before the advent of a nervous system) possess both a system of action and a double form of sensors (proprioceptive and others). Thus, we may venture to suggest that the hypothesis we develop here, which is valid for complex perceptual systems, actually corresponds to a mechanism which is much more general and which is common to all forms of life as they exist from the unicellular scale onward (Iscla and Blount, 2012; Lebois et al., 2012). Thus life, in its primary organization, never exists in a pure feed-forward mode; pure effectuation does not seem to exist; this is in the end compatible with the circular forms of organization characteristic of the later cybernetic approaches. It remains to launch an enquiry into the genesis of the sensor/effector partition in the course of the advent of life itself.

On this basis, and in coherent fashion at the theoretical level, we are led to formulate the three following points:

(1) With reference to the theory of autopoiesis, the paradigm of enaction poses that cognition implies an organization that is proper to living organisms.

(2) This organization, which involves a characteristic circularity at the level of metabolism (a network of elements which produce the elements necessary for its own functioning), replays the scheme of circularity at the nascent sensory-motor level in terms of the relation between effectors and sensors.

(3) The notion of action as pure effectuation no longer having a place (outside the context of the mechanistic conception of automata), it could give way to the concept of enaction which is more favorable to an effort at the characterization of the organization and the lived experience of living and thinking agents.

By way of conclusion, it appears that the next theoretical step will aim at developing the conception of a form of enactive memory which escapes from the bounds of current coupling, without reducing it to a simple representation that can be activated on an occasional basis. Such a memory could be the basis for justifying the appearance of the self and the world.

## Conflict of Interest Statement

The author declares that the research was conducted in the absence of any commercial or financial relationships that could be construed as a potential conflict of interest.

## Footnotes

1 This generic term designates any mechanism which performs the conversion of a signal of one sort into an equivalent signal of another sort. Thus, any sort of sensory organ (photoreceptor, semi-circular canal, or whatever) performs a transduction, which is different for each of them.

2 Although the term “sensory-motor” is more frequent in the literature, we prefer here the inverse formula, besides having the merit of emphasizing the primacy of the action, it also affirms both its role in producing variation in the sensory input, and the importance of the agency of the movement.
